# The Dutch National TissueArchive Portal enables efficient, consistent, and transparent procurement of diagnostic tissue samples for scientific use

**DOI:** 10.1007/s10561-021-09949-1

**Published:** 2021-08-25

**Authors:** Robin Verjans, Annette H. Bruggink, Robby Kibbelaar, Jos Bart, Aletta Debernardi, Tieneke B. M. Schaaij-Visser, Stefan M. Willems, Folkert J. Van Kemenade

**Affiliations:** 1 BBMRI.nl, Lygature, Utrecht, The Netherlands; 2 The Nationwide Network and Registry of Histopathology and Cytopathology in the Netherlands (PALGA), Houten, The Netherlands; 3 Pathology Friesland, Leeuwarden, The Netherlands; 4 Department of Pathology and Medical Biology, University Medical Center Groningen, University of Groningen, Groningen, The Netherlands; 5 Netherlands Society for Pathology (NVVP), Leiden, The Netherlands; 6 Department of Pathology, Erasmus University Medical Centre, Rotterdam, The Netherlands

**Keywords:** Biobank, Biobank information technology, FFPE material, Personalised medicine

## Abstract

Biobanks play a crucial role in enabling biomedical research by facilitating scientific use of valuable human biomaterials. The PALGA foundation—a nationwide network and registry of histo- and cytopathology in the Netherlands—was established to promote the provision of data within and between pathology departments, and to make the resulting knowledge available for healthcare. Apart from the pathology data, we aimed to utilize PALGA’s nationwide network to find and access the rich wealth of Formalin-Fixed Paraffin-Embedded (FFPE) tissue samples for scientific use. We implemented the Dutch National TissueArchive Portal (DNTP) to utilize PALGA’s nationwide network for requesting FFPE tissue samples. The DNTP consists of (1) a centrally organized internet portal to improve the assessing, processing, harmonization, and monitoring of the procurement process, while (2) dedicated HUB-employees provide practical support at peripheral pathology departments. Since incorporation of the DNTP, both the number of filed requests for FFPE tissue samples and the amount of HUB-mediated support increased 55 and 29% respectively. In line, the sample procurement duration time decreased significantly (− 47%). These findings indicate that implementation of the DNTP improved the frequency, efficiency, and transparency of FFPE tissue sample procurement for research in the Netherlands. To conclude, the need for biological resources is growing persistently to enable precision medicine. Here, we access PALGA’s national, pathology network by implementation of the DNTP to allow for efficient, consistent, and transparent exchange of FFPE tissue samples for research across the Netherlands.

## Introduction

Biobanks play an essential role in enabling biomedical research by facilitating the collection, processing, storing, and managing of valuable human biological resources (including cells, tissues, or bodily fluids) and related clinical data (Coppola et al. 2019; Zohouri and Ghaderi 2020). At its best, biobank research has the potential to increase the understanding of underlying disease mechanisms, allow for biomarker detection, and advance drug discovery in the current era of personalised medicine. Various prerequisites must be fulfilled to maximize the potential of biobank research, including: (1) standardization and harmonization of biobank resource management and procurement, (2) detailed information exchange along the entire biobanking process, and (3) compliance to the FAIR (Findable, Accessible, Interoperable, and Reusable)-principles (Barnes and Watson 2020; Kinkorova and Topolcan 2018; Wilkinson et al. 2016). Implementation of a governance addressing all these requirements simultaneously to ensure an efficient, consistent, and transparent exchange is challenging.

Therefore, research infrastructures have been implemented with the aim to connect players in the biobanking field—researchers, biobankers, industry, patients, and subjects—to enable multi-centre research and to utilize their associated clinical records to enhance the efficiency of the healthcare system for the care of patients and the support of researchers. Nationwide biobanking research infrastructures have been implemented in different countries across Europe, including the UK (Yuille et al. 2010), Sweden (Perskvist et al. 2015; Zagai et al. 2019), Switzerland (Mooser and Currat 2014), Italy (Carotenuto et al. 2015), Belgium (Linsen et al. 2019; Van Den Heuvel et al. 2020), and Finland (Vesterinen et al. 2020). In the Netherlands, already in 1971, the PALGA foundation was established to connect pathology departments across the country to establish a national, pathology registry to facilitate communication and information exchange within and between pathology departments, and to use the resulting knowledge for healthcare. The initial focus of PALGA was to allow pathologists to find all relevant data in only one country-wide database, regardless of the hospital of admission of the patient. In addition to the pathology data, the nationwide network established by PALGA created the possibility to find and access the rich wealth of the accompanying Formalin-Fixed Paraffin-Embedded (FFPE) tissue samples, archived at and administrated by the local pathology departments. PALGA can function as a ‘secondary-use’ biobank of all pathological excerpts and materials, meaning that these resources were collected by pathologists with the primary goal of enabling diagnosis within a patient care setting. Importantly, pathology data and FFPE tissue samples do not only form a fundamental source for decision making on an individual patient level but can also be utilized for studying disease on a population basis (Talu et al. 2020).

Therefore, next to facilitating the exchange of data for healthcare, we aimed to utilize PALGA’s network as an intermediate between researchers and the pathology departments to find and access a federated, national archive for requesting FFPE tissue samples for research. Initially, PALGA already provided information on which department stored the FFPE tissue samples of interest with the corresponding contact information, allowing the scientist to collect these for research. However, this past process of FFPE material procurement was characterized by a lengthy duration, a lack of transparency, and inconsistent compliance to privacy protection and legal regulations, all hindering the exchange for research.

The existent PALGA pathology database was enriched by addition of the Dutch National TissueArchive Portal (DNTP) to use PALGA’s nationwide pathology network to establish an efficient, consistent, and transparent FFPE tissue sample procurement process in the Netherlands. This project was initiated by BBMRI.nl, PALGA, and the Netherlands Society for Pathology (NVVP). The governance of the DNTP is organized as follows: the PALGA foundation traditionally managed a national, nationwide pathology database. Jointly with PALGA, software tools were developed to both advance the requests for data as well as facilitate tissue sample procurement. The BBRMI.nl project installed HUB-employees, oversaw the backlog for the software, allocated the budget, and built the DNTP IT application focusing on tissue sample procurement, while jointly with PALGA a request tool for data accessibility was made. For stakeholder alignment, PALGA, the NVVP, and BBMRI.nl communicated to laboratories and science representatives. A budget of €1.2 m was allocated for software of the PALGA Portal (40%), hosting (10%), HUB-employees (10%), and program and software managers (40%). As of 2015, PALGA performed the extension, maintenance, and hosting of the tool that will be updated in 2021 in close collaboration with a BBMRI.nl sequel project. The DNTP consists of a centrally organized IT application and its complementary local HUB-mediated support for logistical processing. This paper describes the previous landscape of challenges that hindered the findability and utilization of the FFPE tissue sample collection and how we set-up efficient, consistent, and transparent exchange of FFPE tissue samples by implementation of the DNTP infrastructure, and the motifs behind it.

**Table:** 

Information Box 1: PALGA foundation	
When PALGA was founded in 1971, its goal was to advance communication within and between the pathological departments in the Netherlands to improve diagnosis (by utilizing information from previous procedures and potentially avoiding doubling of procedures) at an individual level and, eventually, disseminate its aggregated knowledge to healthcare. The system gained nationwide coverage in 1991, providing a daily fully automated update of diagnoses with mesh terms for queries. Nowadays, PALGA includes all (43) pathological departments in the Netherlands, has a national archive for all pathological excerpts (> 77 million) and provides access to FFPE tissue samples (> 40 million) deriving from observational research. PALGA’s federated pathology collection continues to grow with more than 2 million excerpts per year. Requested samples include mainly FFPE tissue materials and its corresponding pathological data. A more detailed description of the initial infrastructure of PALGA has been described (Casparie et al. 2007) and can be found online (www.palga.nl)	

## DNTP combines a central organisation with local support

The DNTP was designed to stimulate the use of the countrywide, but de-centrally located FFPE material archives by improving the efficiency, consistency, and transparency of its procurement process. These aims highlight the need for a nationwide overarching infrastructure to harmonize the procurement procedures between the various local pathology departments. In addition, detailed information exchange between all involved players (pathologists, researchers, biobanking personnel, etc.) can promote insight into and compliance to both ethical and legal regulations. While doing so, tailored, local support needs to be provided to optimize the procurement process for the researcher. In order to achieve these goals, specific objectives were established:

1. Improve the efficiency of the FFPE material procurement and navigation by defining a central, overarching strategy.
2. Provide practical, tailored support on the overarching strategy at a local level during the entire FFPE material procurement process.
3. Increase the transparency of FFPE material exchange through improved monitoring (track and trace) of its procedure.
4. Stimulate the use of FFPE tissue samples for research by making the federated database and archive accessible for all involved stakeholders across the Netherlands.
5. Facilitate General Data Protection Regulation (GDPR) compliance through improved registration of data and material requests.
6. Increase probability of proper return of FFPE material to the original pathology department.

**Fig. 1:**
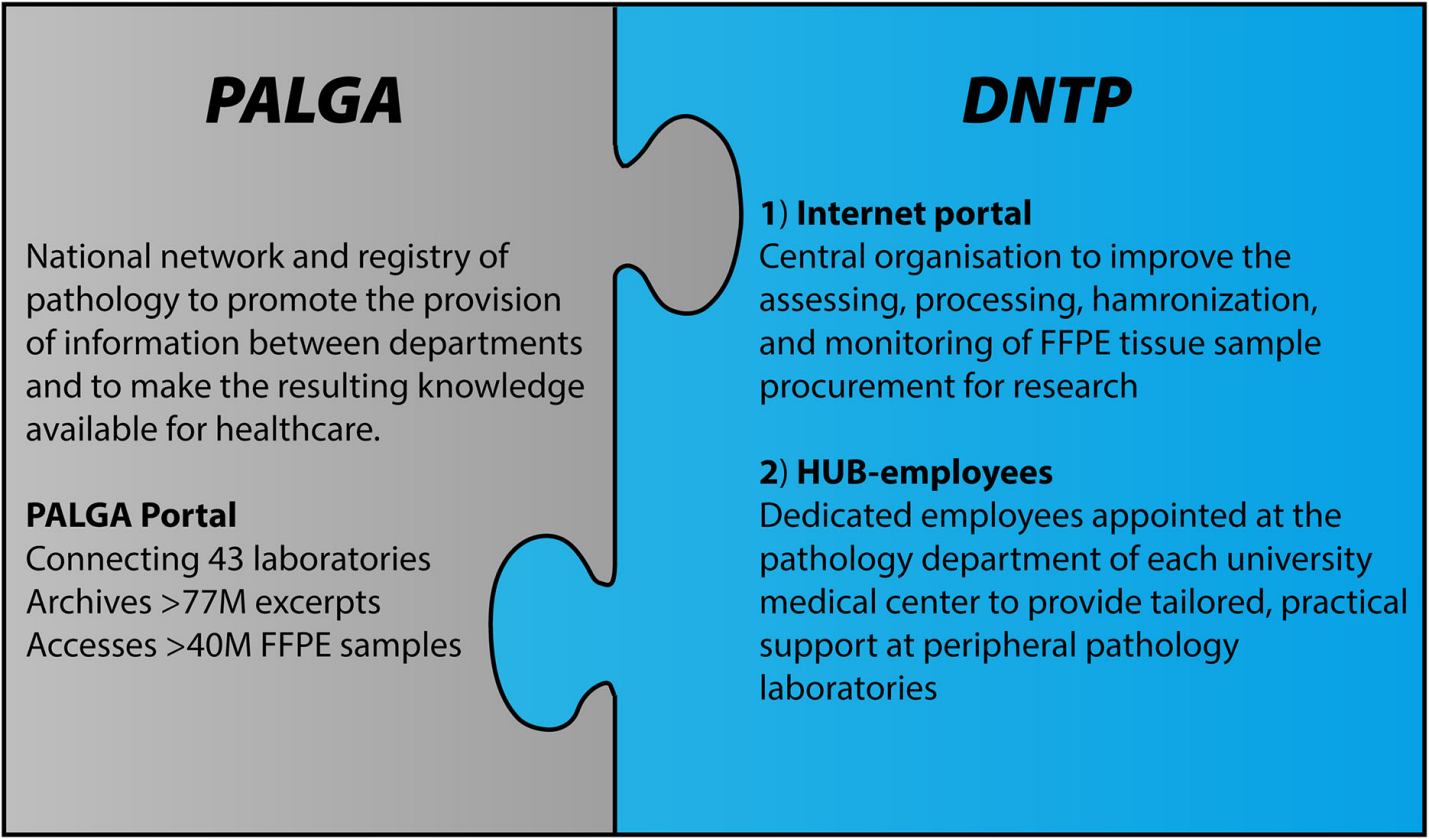
Schematic representation of the implemented expansion of the existent PALGA infrastructure with the DNTP, consisting of an internet portal and HUB-employee-mediated support

The strategic decision was made to design the DNTP infrastructure as a unique combination of (1) a centrally organised IT software application for assessing, processing, harmonizing, and monitoring of FFPE sample procurement requests, complemented with (2) local, tailored support provided by, so called, HUB-employees for assistance in procurement of the requested samples while overseeing and controlling compliance (Fig. 1).

### Centralised IT application for assessing, processing, harmonizing, and monitoring of FFPE procurement

A sustainable governance structure is indispensable to make optimal use of the rich collection of biobank-deriving biomaterials and its associated data. Information technology (IT) applications have been developed to address the biobanking processes, optimize workflow efficiency, and provide quality assurance (Boutin et al. 2016; Dangl et al. 2010; Mate et al. 2017; Proynova et al. 2017). Here, we created a ‘one-stop shop’-internet portal as a central overarching infrastructure to improve the assessing, processing, harmonization, and monitoring of the FFPE tissue sample procurement process for both requesters and providers across the Netherlands.

Researchers can request specific FFPE tissue samples from pathology departments across the Netherlands by filing a request using the internet portal (https://aanvraag.palga.nl/). Both academic and commercial researchers can be granted access to the collections of FFPE samples. Each application is reviewed on scientific quality and compliance to privacy regulations by the review committee of the corresponding pathology department. The composition of the review committees of the providing laboratories can differ between pathology departments, including amongst other pathologists, scientists, as well as legal and ethical experts. This reviewing process takes place on a continuous basis to guarantee timely response towards the applicant. The application review process requires that the researcher specifies relevant institutional information, including the associated project leader and pathologist to allow PALGA to verify the researchers’ association with the scientific institute. More importantly, the researcher is obligated in this request proposal to specify the type and number of FFPE material needed. The filed request will then be approved by the corresponding department(s). In the case that the request proposal is assessed as insufficient, the researcher is informed and will be asked to modify their proposal to address the concerns raised. In case of a positive evaluation of the request, the researcher and the local pathology department will make further agreements for use of the material. FFPE tissue samples may be used for scientific research only. Financial expenses may be charged, varying between pathology departments, to cover the costs needed for collection, handling, and storage of the FFPE tissue samples. Procurement of the samples can then be initiated according to the terms of the agreement.

Thus, the main improvements due to renewed incorporation of the centrally organised IT application software are:

1. Increased accessibility to the federated archive of FFPE biomaterials for all stakeholders located across the Netherlands.
2. One request involving samples derived from multiple pathology departments located across the Netherlands, can be communicated and processed with one query.
3. Up-to-date information exchange concerning FFPE material location, distribution, and return (‘track and trace’).
4. Facilitate adherence to the legal, ethical and privacy framework due to improved documentation.

Although extension of the existing PALGA architecture with the internet portal helped to define a centrally organised FFPE tissue sample procurement procedure, practical support at the pathology departments was crucial and needed fostering. Therefore, a second strategic part was developed to harmonize implementation of the centrally organised procedures and to allow for practical support on a local level.

### HUB-employees provide local, tailored support for FFPE material procurement

The first step of FFPE sample procurement after approval through the internet portal is notification of the corresponding pathology department concerning the request. In the second part of our novel approach, dedicated employees at each pathology department of the eight university medical centres have been appointed part-time to function as so-called HUB-employees. These HUB-employees are responsible for logistical and technical support at peripheral pathology departments. Currently, eight HUB-employees are employed which provide support at their own university medical centre and, on average, an additional two to three connected non-UMC pathological centres. The HUB-employees perform a wide range of activities depending on the specific request and the pathology department involved. HUB-employees consult the requestor prior as well as during the entire request-, procurement-, and retrieval process. In addition, at the PALGA office, we have installed a so-called national HUB information point. This central PALGA-associated HUB-employee can negotiate requests in order to optimize queries. HUB-employees can take the responsibility to locate the samples in the storage of their university medical centre as well as for the oversight of the procurement in the connected institutes. Once the requested samples have been collected, quantity (available tissue, slides, stainings, etc.) will be determined for each individual sample. It is the responsibility of the HUB-employee to ensure preservation of sufficient amount of tissue to prevent compromising the ability to conduct additional clinical test for diagnosis of the patient. In order to come to a valid decision, the HUB-employee considers the lab techniques performed on the requested sample. When uncertainty exists if sufficient amount of tissue is available, the HUB-employee prioritizes preservation of the tissue and will contact the applicant. Notably, HUB-employees are in most cases either lab technicians that know the tissue limits of e.g., biopsies or have access to expertise in this field. In the majority of the requests, quantity is in accordance with the filed agreement and the requested samples will be sent to the researcher. The researcher can confirm receipt of the FFPE tissue samples by using the online IT application software. Importantly, the DNTP also allows for monitoring of timely return of the FFPE tissue samples by the researcher to the respective department.

The HUB-mediated local, logistical support on FFPE tissue sample procurement has several advantages:

1. Provision of practical support service for collecting and processing FFPE samples in a hub-in-spoke approach.
2. Increased follow-up and traceability of FFPE tissue sample requests.
3. Relieve of the workload experienced by individual pathologists in the respective departments that helped in procurement of FFPE materials in accordance with the highest standards of stewardship, while formally not being assigned with these tasks.
4. Active engagement in the pathology community within the Netherlands, increasing the mutual responsibility to maximize the use of pathological samples and data.
5. Assurance of an organisation continuity in sample procurement for this biobank.

A pilot use-case was performed to assess the advantages of the DNTP during the HUB-employee-mediated tissue procurement process of PALGA’s collection (the so-called Rainbow 7 project). The overall aim of this project was to enrich existing Dutch cohort studies with FFPE tumor tissue samples to create tissue microarrays (TMAs) and tumor-DNA, to enable molecular pathological epidemiology and biomarker development in large cohort studies. In total, 11,706 cancer cases with tumors in one of seven different sites of interest (oesophagus, gastric, colorectal, pancreas, breast, ovary, bladder) were selected to request archived diagnostic FFPE samples from. For each individual case, if available, 2 tumor tissue FFPE blocks and 1 healthy tissue FFPE block were collected. Because this was a nationwide project concerning 43 pathology departments throughout the Netherlands, regional phases of collection were planned and executed by the HUB-employees. The HUB-employee system allowed for an efficient collection in which each donor department was visited only once to collect all tumor tissues and cohorts combined. Tumor tissue samples were retrieved successfully for 9350 cases (83 oesophagus, 107 gastric, 3579 colorectal, 71 pancreas, 3867 breast, 611 ovary, 1032 bladder). This represents 79.9% of the 11,706 requested blocks. Healthy tissue blocks were retrieved for 5962 cases, which is 50.9% of the 11,706 requested blocks. TMA blocks were produced in duplicate to facilitate use of precious material by external researchers. For these cases, also tissue sections for DNA-extraction were produced. After completion of the TMA-production and DNA-section process, all collected original blocks have been returned to the respective 43 donor departments. To conclude, this use-case supports that implementation of the DNTP ensures harmonization, compliance, and quality management along the entire tissue procurement process on a nationwide scale.

**Fig. 2:**
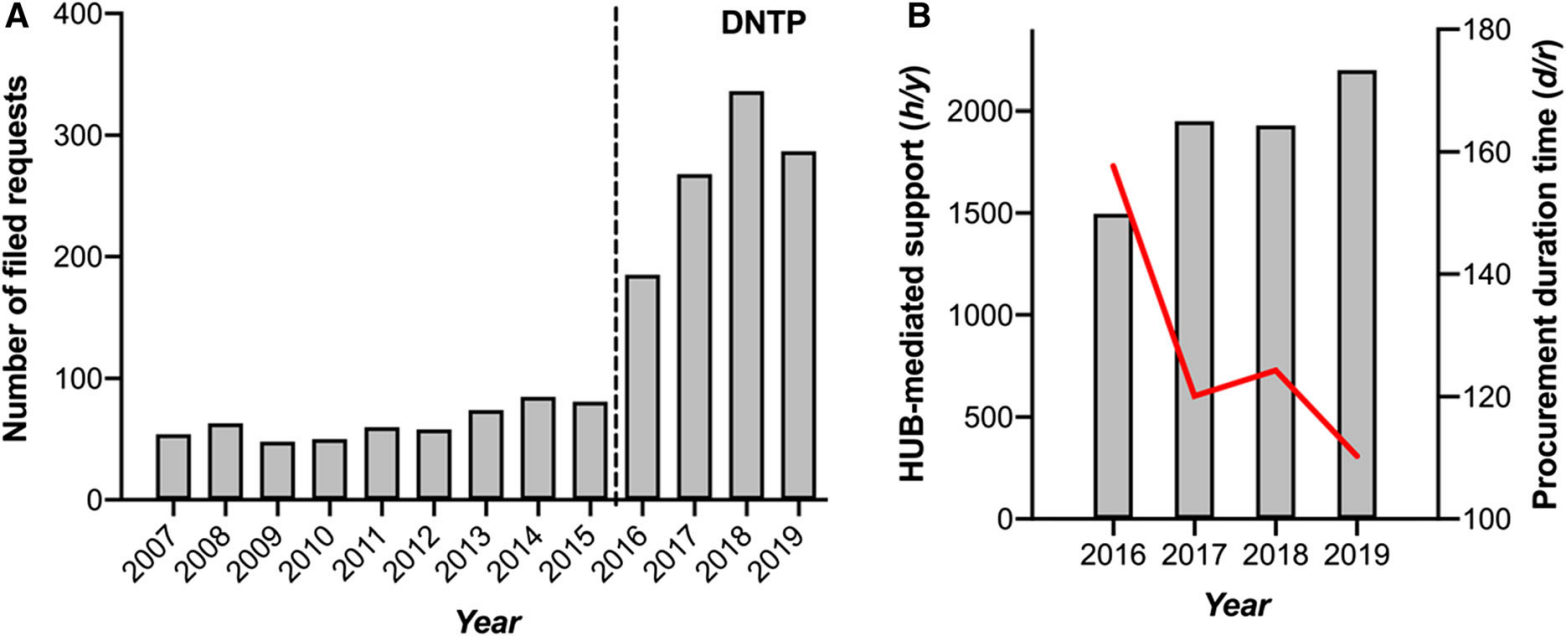
Implementation of the DNTP improved the frequency, efficiency, and transparency of FFPE tissue sample procurement for research in the Netherlands. **a** The number of filed requests for FFPE tissue samples prior and after DNTP implementation in 2016. **b** The amount of performed HUB-mediated support in hours per year (represented by the bar graphs) and the average procurement duration time per request in days (represented by the red line) during the years following implementation

Following the use-case, the performance of the DNTP was evaluated over time, as indicated by the number of filed requests, the amount of provided HUB-mediated support, and the sample procurement duration time (Fig. 2). The sample duration time is defined as the time between the moment that the corresponding pathology department is notified of the request and ends when the HUB-employee has issued all requested samples. Directly upon implementation of the DNTP in 2016, the number of filed requests by using the DNTP showed a strong increase to 185 (+ 117%) from 81 in the year prior (Fig. 2a). During the four years following DNTP implementation 269 requests were filed on average per year while during the period of 2007 till 2015 the average number of filed requests was only 64 per year (Fig. 2a). In line, the time invested by HUB-employees to provide support increased from 1496 h in 2016 to 2201 h (+ 47%) in 2019 (Fig. 2b). Only upon the moment of DNTP implementation, quantification of the duration time of sample procurement was possible. The mean sample procurement duration time per request consistently decreased from 157.7 days in 2016 to 110.3 days (− 43%) in 2019 (Fig. 2b). Taken together, these findings indicate that implementation of the DNTP improved the frequency, efficiency, and transparency of FFPE tissue sample procurement for research in the Netherlands.

## Discussion and conclusions

The need for large number of biological samples and its associated data is growing persistently to enable the development of precision medicines. In order to achieve this goal, researchers should have secure access to high-quality biological materials and data deriving from large patient populations. This demand can only be met when secondary or patient-derived-biobanks function on a (inter)national scale, driving biobanks to innovate their infrastructures to allow for optimal governance and increase utility of these collections.

Here, we describe how we implement the DNTP to utilize PALGA’s network as an intermediate to find and access the national, federated FFPE tissue sample archive for efficient, consistent, and transparent FFPE tissue sample procurement. “The existence of PALGA as a nationwide network and registry of histo- and cytopathology in the Netherlands was of indispensable value for the design and development of the DNTP infrastructure to find and access an ever-growing collection of archived FFPE tissue samples. Importantly, access to the rich source of tissue samples would not have been possible without PALGA functioning as an intermediate between researchers and the pathology departments. Alternatively, but much more limited, one can envisage a cancer registry to start with a cancer block procurement organization if e.g., an organization like PALGA lacks in jurisdictions.” The DNTP includes a centralised online IT application as an overarching infrastructure to improve the assessing, processing, harmonization, and monitoring of the FFPE tissue specimen procurement process. Practical support while implementing this overarching strategy at the various pathology departments was achieved through appointing dedicated HUB-employees in a hub-in-spoke approach. This dual approach allows for efficient, consistent, and transparent exchange of FFPE tissue samples for healthcare decision-making as well as for research across the Netherlands. Moreover, the improved infrastructure and communication between the various pathology departments introduces a feeling of mutual responsibility over the optimal use of histopathological samples and data on a national scale.

This mutual responsibility is mirrored by the European Biobanking and BioMolecular resources Research Infrastructure—European Research Infrastructure Consortium (BBMRI-ERIC) (Holub et al. 2016; Litton 2018; van Ommen et al. 2015), which was founded to coordinate biobanking-related activities and governance. The goal of the BBMRI-ERIC infrastructure is to link biobanks across Europe to foster cooperation and research that benefits both patients and European citizens. It does so by electing national nodes in one of its 16 member countries (Holub et al. 2016; Litton 2018; van Ommen et al. 2015). In the Netherlands, PALGA collaborates within the national node BBMRI.nl to maximize the use of biomaterials and data findable, accessible, and usable for health research on the prevention, diagnosis, and treatment of diseases.

Monitoring of key performance indicators of FFPE tissue sample procurement for research in the Netherlands only became possible with the implementation of the DNTP. Evaluation of these key performance indicators indicate that its implementation improved the frequency, efficiency, and transparency of FFPE tissue sample procurement for research in the Netherlands. However, the large increase in the number of filed requests for FFPE tissue samples since the incorporation of the DNTP cannot solely be attributed to an increased utility of FFPE tissue samples for research. Previous exchange of FFPE tissue samples through personal contact between researchers and pathologists has moved from an undocumented situation to registered transfer via the DNTP portal. Although great progression in monitoring been made, FFPE tissue sample exchange circumventing the DNTP should be minimized to increase tracking of biomaterials to allow for compliance the ethical and legal regulations. Reporting of other key performance indicators, such as the procurement duration time, depends on information provided by the researcher. Obtaining inaccurate or incomplete information may lead to misinterpretation of DNTP performance.

Extension of PALGA with the DNTP has increased the utility of the federated archive and optimized FFPE tissue sample procurement procedures but necessitates continuous innovation to further improve. Future developments may include, first, nationally standardized procedures between the requestor and provider specified for each request approval procedure, defining: (A) standards regarding the type of FFPE tissue samples (tissue blocks versus—sections and its metadata) provided and the expenses charged (e.g., fee-for-service agreements to cover the costs), and (B) nationally standardized material transfer agreements (MTA). The latter can be achieved by using an MTA template that has been set up in conjunction with legal advisors of all the Dutch academic hospitals. This MTA template serves to facilitate and regulate the transfer of human materials that were previously collected by a Dutch University Hospital from patients, participants, and/or volunteers for the purpose of scientific research (see https://www.elsi.health-ri.nl/ for more information).

Second, future DNTP developments aim to enable regular informing of pathology departments concerning the number of processed requests for FFPE tissue samples and the resulting scientific output. Insight for the individual pathology departments into their contribution and the scientific output resulting from the procurement process, with or without the help of HUB-employees, can be used for internal/external documentation and is key in maintaining engagement.

Third, we ultimately aim to incorporate access to the DNTP into a comprehensive internet portal for researchers, that goes beyond the FFPE tissue samples, and provides all available data, samples, images, etc. from various repositories, biobanks, and registries in the Netherlands for research. One example of such an IT application in the process of development that aims to provide in an all-encompassing plethora of data is the Podium service of the Health-RI initiative (see https://www.health-ri.nl/services/podium). This would simplify the researcher experience to use only one internet portal to find, access, and request various types of biomaterials and data deriving from a plethora of different pathologies. Furthermore, this would allow the DNTP to contribute as a FAIR-source for the continuous development of multi-disciplinary, multi-centre research to drive personalised medicine (Abul-Husn and Kenny 2019). Importantly, compliance of the DNTP to the FAIR-principles is required to offer a truly connected infrastructure that supports widespread access (Proynova et al. 2017).

In addition to the technical and logistical developments of the DNTP, substantial efforts have been made to stimulate all stakeholders involved (pathologists, researchers, principal investigators, technical support, managers, etc.) to contribute to this common objective and to align the processes at the various pathology departments. Two roadshows have been organised in which most pathology laboratories and departments have been visited to inventory the requirements to establish the DNTP infrastructure. During these roadshows, the added benefit of HUB-mediated support—being pro bono provision of expert knowledge and competences to alleviate the pathology departments of non-core obligations—was emphasized. Pathologists in the laboratories have been pointed out to the possibility of group accounting for publications (as designed by PALGA) to give them credit for their role in maintaining access of tissues for science. Moreover, pathologists and HUB-employees are being informed and inspired on a continuous basis via various communication channels, including national pathology congresses, the NVVP magazine, and the HUB- employee annual meeting.

To conclude, implementation of the DNTP infrastructure largely increases utilization of PALGA’s network to find and access the federated, national archive of FFPE tissue samples for efficient, consistent, and transparent sample procurement. The pathologists in the Netherlands aim to harmonize resource management and handling, and foster quality-assurance along the entire biobanking process. This scientific infrastructure allows the vast registry of archived diagnostic tissue and its associated data to be used as a fundamental source for precision-medicine, combining personalized medicine with population health.

## Data Availability

The datasets generated during and/or analysed during the current study are available from the corresponding author on reasonable request.
